# Bioactive Secondary Metabolites from the Culture of the Mangrove-Derived Fungus *Daldinia eschscholtzii* HJ004

**DOI:** 10.3390/md17120710

**Published:** 2019-12-17

**Authors:** Hai-Xia Liao, Tai-Ming Shao, Rong-Qing Mei, Guo-Lei Huang, Xue-Ming Zhou, Cai-Juan Zheng, Chang-Yun Wang

**Affiliations:** 1 Key Laboratory of Tropical Medicinal Resource Chemistry of Ministry of Education, Hainan Normal University, Haikou 571158, China; m17864275062@163.com (H.-X.L.); 15707973425@163.com (R.-Q.M.); tianleilei526@163.com (G.-L.H.); xueming2009211@126.com (X.-M.Z.); 2 Key Laboratory of Tropical Medicinal Plant Chemistry of Hainan Province, College of Chemistry and Chemical Engineering, Hainan Normal University, Haikou 571158, China; 3 Laboratory for Marine Drugs and Bioproducts, Qingdao National Laboratory for Marine Science and Technology, Qingdao 266071, China; 4 Guangxi Key Laboratory of Agricultural Resources Chemistry and Biotechnology, College of Chemistry and Food Science, Yulin Normal University, Yulin 537000, China; shaotm1689@163.com

**Keywords:** *Daldinia eschscholtzii*, polyketides, naphthoquinones, antibacterial activity, *α*-glucosidase inhibitory activity

## Abstract

Two new polyketides, 8-*O*-methylnodulisporin F (**1**) and nodulisporin H (**2**), two new naphthoquinones, 5-hydroxy-2-methoxy-6,7-dimethyl-1,4-naphthoquinone (**3**) and 5-hydroxy-2-methoxynaphtho[9–*c*]furan-1,4-dione (**4**), and a new naphthofuran 1,3,8-trimethoxynaphtho[9–*c*]furan (**5**), along with five known compounds 4-*O*-methyl eleutherol (**6**), 2-acetyl-7-methoxybenzofuran (**7**), (-)-orthosporin (**8**), diaporthin (**9**), and 6-hydroxymellein (**10**), were obtained from the EtOAc extract of the mangrove-derived fungus *Daldinia eschscholtzii* HJ004. The structures of the isolated compounds were elucidated by extensive NMR and MS analyses, while the absolute configurations of the stereogenic carbons were established based on experimental and calculated electronic circular dichroism spectra. Compounds **4** and **7** displayed a potent inhibitory activity against *α*-glucosidase with the IC_50_ values of 5.7 and 1.1 μg/mL, respectively. Compounds **1** and **2** showed a moderate antibacterial activity against *Staphylococcus aureus*, methicillin-resistant *S. aureus* (MRSA) and *Bacillus cereus*, with minimum inhibitory concentration (MIC) values ranging from 6.25 to 12.5 μg/mL. Compound **3** exhibited antibacterial activity against *B. cereus* with the MIC value of 12.5 μg/mL.

## 1. Introduction

Diabetes poses a serious threat to cardiovascular diseases, and is the eleventh common cause of disability worldwide. According to the 8th edition of the International Diabetes Federation Diabetes Atlas 2017, more than 425 million people worldwide are suffering from diabetes, and China alone accounts for one-third of that [1,2]. Around 90% of all cases of diabetes were Type 2 diabetes mellitus [3,4,5]. *α*-Glucosidase, a crucial enzyme that breaks down complex carbohydrates for absorption, plays a key role in the treatment of diabetes. *α*-Glucosidase inhibitors such as natural products acarbose and voglibose can reduce the impact of carbohydrates on blood glucose level and prevent the digestion of carbohydrates, indicating that natural compounds play an important role in a discovery of anti-diabetic drugs [6,7].

**Figure 1:**
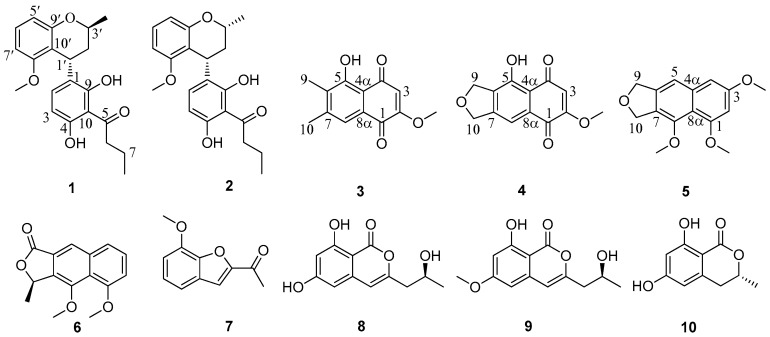
Chemical structures of **1**–**10**.

Mangrove-derived fungi can produce secondary metabolites with novel structures and biological activities [8]. In our ongoing research on mangrove-derived fungi, we have found some new bioactive metabolites (including metabolites with inhibitory activity against *α*-glucosidase) [9,10,11,12,13,14]. A mangrove-derived fungus *Daldinia eschscholtzii* HJ004 attracted our attention due to the inhibitory activity against *α*-glucosidase of its EtOAc extract, and three polyketide derivatives with strong inhibitory activity against *α*-glucosidase have been isolated [9]. A further chemical investigation of the fermentation broth resulted in the identification of two new polyketides 8-*O*-methylnodulisporin F (**1**) [15] and nodulisporin H (**2**) [15], two new naphthoquinones 5-hydroxy-2-methoxy-6,7-dimethyl-1,4-naphthoquinone (**3**), and 5-hydroxy-2-methoxynaphtho[9–*c*]furan-1,4-dione (**4**), and a new naphthofuran 1,3,8-trimethoxynaphtho [9–*c*] furan (**5**), together with five known compounds, 4-*O*-methyl eleutherol (**6**) [16], 2-acetyl-7-methoxybenzofuran (**7**) [17], (-)-orthosporin (**8**) [18], diaporthin (**9**) [18], and 6-hydroxymellein (**10**) [19] (Figure 1). The electronic circular dichroism (ECD) calculation method was used to establish the absolute configurations of the stereogenic carbons in **1** and **2**. Herein we report the isolation, structure elucidation, antibacterial activity, and inhibitory activity against *α*-glucosidase of these compounds.

## 2. Results and Discussion

**Figure 2:**
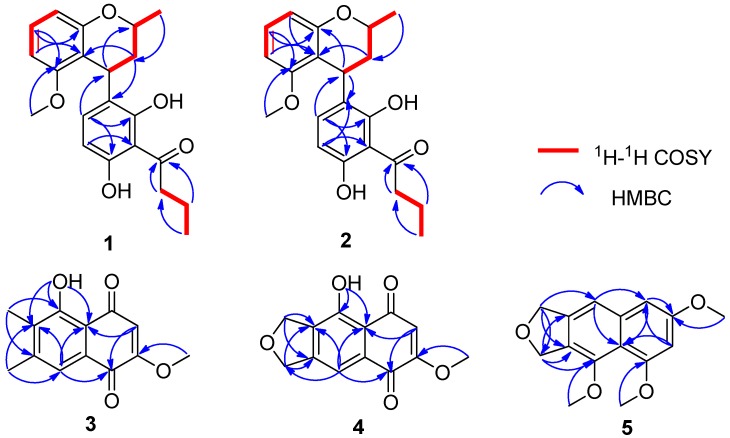
Key ^1^H-^1^H COSY and HMBC correlations in **1**–**5**.

Compound **1** was isolated as a white amorphous powder. Its molecular formula C_21_H_24_O_5_ (ten degrees of unsaturation) was determined by the [+]-HR-ESI-MS ion peak at *m*/*z* 357.1693 [M + H]^+^ (calcd. for 357.1697, C_21_H_25_O_5_) (Figure S8). The ^1^H NMR spectrum (Figure S1) of **1** indicated the presence of five aromatic protons at *δ*_H_ 7.14 (dd, *J* = 8.0, 7.8 Hz), 6.64 (d, *J* = 8.0 Hz), 6.55 (d, *J* = 8.0 Hz), 6.42 (d, *J* = 7.8 Hz), and 6.07 (d, *J* = 8.0 Hz), and a methoxyl signal at *δ*_H_ 3.63 (s). In addition, in the sp^3^ region of the ^1^H NMR spectrum, with the help of DEPT-135 and HMQC spectra (Figures S1, S3 and S4), two methine protons at *δ*_H_ 4.54 (dd, *J* = 5.2, 0.8 Hz) and 3.98 (ddq, *J* = 11.6, 6.4, 2.0 Hz), three methylenes at *δ*_H_ 3.12 (t, *J* = 7.2 Hz), (2.07 (dt, *J* = 14.0, 2.0 Hz), 1.89 (ddd, *J* = 14.0, 11.6, 5.2 Hz)), and 1.75 (m), and two methyls at *δ*_H_ 1.30 (d, *J* = 6.4 Hz) and 1.01 (t, *J* = 7.4 Hz) were observed, respectively. The ^13^C NMR spectrum showed 21 carbon signals, which, in combination with DEPT-135 and HSQC spectra (Figures S2–S4), can be categorized as three methyls (*δ*_C_ 55.8, 21.5, and 14.1), three methylene sp^3^ (*δ*_C_ 46.8, 34.5, and 17.9), two methine sp^3^ (δ_C_ 67.9 and 29.5), five methine sp^2^ (δ_C_ 135.3, 128.1, 109.7, 105.4, and 102.2), seven non-protonated sp^2^ (*δ*_C_ 161.0, 158.1, 156.8, 156.2, 126.0, 111.7, and 109.5) and one carbonyl (*δ*_C_ 208.1) carbons. The above NMR data suggested that the structure of **1** was very similar to that of nodulisporin F [20] except for the presence of the methoxyl group (*δ*_H_ 3.63/*δ*_C_ 55.7). That the methoxyl group was located at C-8′ was confirmed by the HMBC correlation from 8′-OMe (*δ*_H_ 3.63) to C-8′ (*δ*_C_ 158.1) and the NOESY correlation from H-7′ (*δ*_H_ 6.42) to 8′-OMe (Figures S6 and S7). The 2D NMR data allowed to elucidate the complete planar structure of **1** (Figure 2 and Figures S4–S6).

The relative configurations of the C-1′ and C-3′ estereogenic centers were deduced by the coupling constants (*J*_1′,2′a_ = 5.2 Hz and *J*_3′,2′a_ = 11.6 Hz) in the ^1^H NMR spectrum. The small coupling constant of 5.2 Hz between H-1′ and H-2′a established that H-1′ was in equatorial orientation. By contrast, the coupling constant for *J*_3′,2′a_ = 11.6 Hz indicated the axial orientation of H-3′, and the equatorial position of the Me-3′. These results implied that H-1′ and H-3′ were on the opposite sides. Besides, the NOESY spectrum of **1** showed correlations between H-1′ and H-2′a, as well as between H-3′ and H-2′b (Figure S7). Furthermore, the chemical shifts and coupling constants of H-1′/ H-2′/ H-3′/ H-4′ were close to those of nodulisporin F [20], which suggested a similar configuration. Thus, the relative configurations of **1** were confirmed as 1′R*,3′S*, and **1** was named 8-*O*-methylnodulisporin F.

Compound **2** was also isolated as a white amorphous powder with the same molecular formula as **1**, based on the [+]-HR-ESI-MS ion peak at *m*/*z* 357.1693 [M + H]^+^ (calcd. for 357.1697, C_21_H_25_O_5_) (Figure S16). Its ^1^H- and ^13^C-NMR data closely resembled those of **1**, except for the coupling constants between H-1′ and H-2′ (*J*_1′,2′_ = 10.4, 8.0 Hz) in the ^1^H NMR spectrum (Table 1 and Figure S9). The large coupling constants of *J*_1′,2′a_= 10.4 Hz and *J*_3′,2′a_ = 11.2 Hz confirmed that H-1′ and H-3′ were both in the axial orientation. Additionally, the NOESY spectrum of **2** showed correlations between H-1′ and H-2′b, as well as between H-3′ and H-2′b (Figure S15). These results indicated the relative configurations of **2** as 1′R*,3′R*, and **2** was named as nodulisporin H. Therefore, **1** and **2** are C-3′ epimers.

**Figure 3:**
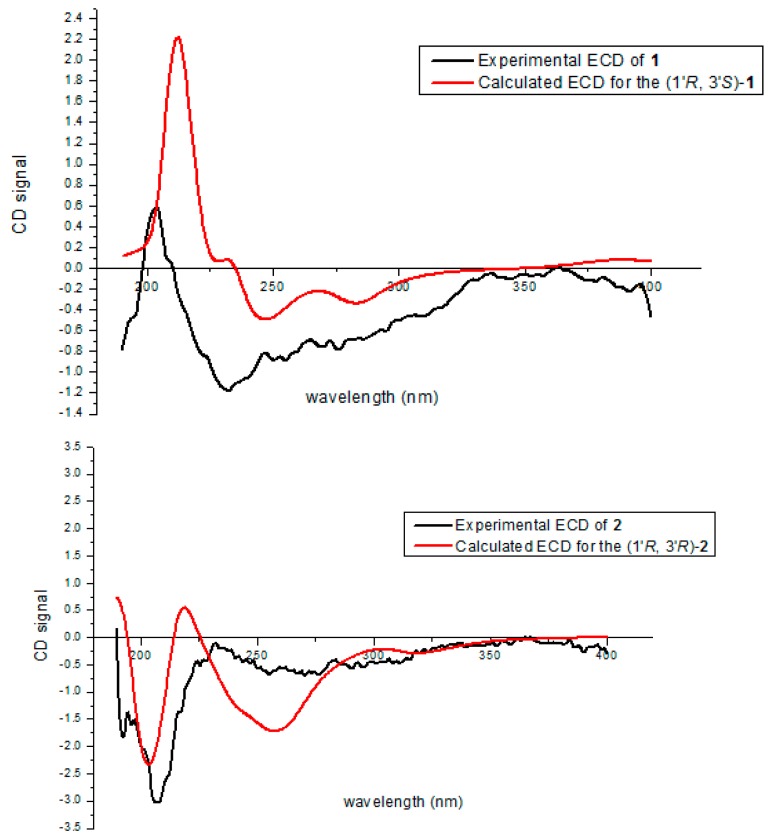
Comparison of experimental and calculated ECD spectra of **1** and **2** in MeOH at the B3LYP/6-311 + G (d, p) level.

The absolute configurations of C-1′ and C-3′ in **1** and **2** were determined by comparison of experimental and calculated ECD spectra. The calculated ECD curve of 1′*R*,3′*S* matched well the experimental ECD curve of **1**, while the calculated ECD spectrum of the 1′*R*,3′*R* matched well the experimental ECD spectrum of **2** (Figure 3).

Compound **3** was obtained as a yellow powder. The [+]-HR-ESI-MS ion peak at *m*/*z* 233.0809 [M + H]^+^ (calcd. for 233.0808, C_13_H_13_O_4_) indicated that the molecular formula of **3** was C_13_H_12_O_4_ (eight degrees of unsaturation) (Figure S21). Its ^1^H NMR data (Table 2) displayed one chelated hydroxyl proton at *δ*_H_ 12.61 (s, OH-5), one aromatic proton at *δ*_H_ 7.50 (s, H-8), one olefinic proton at *δ*_H_ 6.05 (s, H-3), one methoxyl at *δ*_H_ 3.90 (s, OMe-2), and two methyls at *δ*_H_ 2.36 (s, Me-10) and 2.25 (s, Me-9). The ^13^C NMR spectrum, in combination with the HMQC spectrum (Figures S18 and S19), displayed 13 carbon signals, which consisted of two carbonyl carbons at *δ*_C_ 190.9 and 179.8, six non-protonated sp^2^ carbons at *δ*_C_ 161.2, 159.7, 159.7, 145.1, 134.2, and 111.7, two protonated sp^2^ carbons at *δ*_C_ 121.4 and 109.5, one methoxyl carbon at *δ*_C_ 56.7, and two methyl carbons at *δ*_C_ 20.8 and 11.9. The ^1^H and ^13^C NMR data of **3** (Table 2) were very similar to those of 2-methoxy-7-methyljuglone [21]. The only difference was the presence of the methyl group on C-6 in **3**, which was supported by the HMBC correlations from Me-9 to C-5 and C-7. The HMQC and HMBC spectra (Figures S19 and S20) confirmed the structure of **3** (Figure 2) as 5-hydroxy-2-methoxy-6,7-dimethyl-1,4-naphthoquinone.

Compound **4** was obtained as a brown powder, with the molecular formula C_13_H_10_O_5_ (nine degrees of unsaturation) based on the [+]-HR-ESI-MS at 247.0605 [M + H]^+^ (calcd. for 247.0601, C_13_H_11_O_5_) (Figure S26). The ^1^H and ^13^C NMR data of **4** (Table 2) were similar to those of **3**. The obvious difference was that Me-9 (*δ*_H_ 2.25/*δ*_C_ 11.9) and Me-10 (*δ*_H_ 2.36/*δ*_C_ 20.8) in **3** were replaced by two oxymethylene carbons at *δ*_C_ 72.1 (C-9; *δ*_H_ 5.20) and 74.3 (C-10; *δ*_H_ 5.16) in **4**. Combination of the ^1^H and ^13^C NMR data with the molecular formula of **4** indicated the presence of a tetrahydrofuran ring. Therefore, the structure of **4** was established as 5-hydroxy-2-methoxynaphtho [9–*c*] furan-1,4-dione.

Compound **5** was isolated as a yellow powder, with the molecular formula of C_15_H_16_O_4_ (eight degrees of unsaturation) based on [+]-HR-ESI-MS at *m*/*z* 261.1118 [M + H]^+^ (calcd. for 261.1121, C_15_H_17_O_4_) (Figure S31). The ^1^H and ^13^C NMR data (Table 2), assigned with the help of the HMQC spectrum (Figure S29), exhibited the presence of three aromatic protons at *δ*_H_ 7.30 (s, H-5), 6.71 (d, *J* = 2.2 Hz, H-2), and 6.51 (d, *J* = 2.2 Hz, H-4), three methoxyl signals at *δ*_H_ 3.97 (s, OMe-1), 3.89 (s, OMe-3), and 3.84 (s, OMe-8), and two oxymethylene signals at *δ*_H_ 5.26 (s, H-10) and 5.16 (s, H-9). The ^13^C NMR spectrum (Table 2), in combination with the HMQC spectrum (Figures S28 and S29), displayed 15 carbon signals, consisting of seven non-protonated sp^2^ (*δ*_C_ 158.1, 157.5, 150.6, 140.5, 139.0, 127.7, and 115.4), three protonated sp^2^ (*δ*_C_ 114.5, 99.0, and 98.8), two oxymethylene sp^3^ (*δ*_C_ 73.3 and 71.7) and three methoxyl (*δ*_C_ 61.6, 56.2, and 55.4) carbons. The HMBC correlations from H-9 (*δ*_H_ 5.16) to C-5 (*δ*_C_ 114.5), C-7 (*δ*_C_ 140.5) and C-10 (*δ*_C_ 71.7) as well as from H-10 (*δ*_H_ 5.26) to C-6 (*δ*_C_ 127.7) and C-8 (*δ*_C_ 150.6) suggested the presence of a naphthofuran skeleton. Furthermore, the HMBC correlations from OMe-1(*δ*_H_ 3.97) to C-1 (*δ*_C_ 157.5), OMe-3 (*δ*_H_ 3.89) to C-3 (*δ*_C_ 158.1), and OMe-8 (*δ*_H_ 3.84) to C-8 (*δ*_C_ 150.6) established the positions of the three methoxyls at C-1, C-3 and C-8, respectively. Thus, the structure of **5** was elucidated as 1,3,8-trimethoxynaphtho [9–*c*] furan.

Compounds **4** and **7** displayed potent inhibitory activity on *α*-glucosidase with the IC_50_ values of 5.7 and 1.1 μg/mL, respectively. The rest of the isolated compounds exhibited no inhibitory activity against *α*-glucosidase. Acarbose was used as a positive control (IC_50_ = 2.0 μg/mL).

Compounds **1** and **2** showed moderate antibacterial activity against *Staphylococcus aureus*, Methicillin-resistant *S. aureus* MRSA and *Bacillus cereus*, with minimum inhibitory concentration (MIC) values ranging from 6.25 to 12.5 μg/mL (Table 3). Compound **3** exhibited antibacterial activity against *B. cereus* with the MIC value of 12.5 μg/mL (Table 3). The other compounds showed no antibacterial activity against six pathogenic bacteria.

## 3. Materials and Methods

### 3.1. General Experimental Procedures

Both 1D and 2D NMR spectra were measured on a Bruker AV-400 (Bruker Corporation, Fällanden, Switzerland) instrument by using CDCl_3_ as a solvent with TMS as the internal standard. The other experimental procedures were performed as previously described in the literature [9].

### 3.2. Fungal Material

The strain HJ004 was isolated from the stem of mangrove *Brguiera sexangula* var. *rhynchopetala*, collected in the South China Sea, and was identified as *Daldinia eschscholtzii* with the GeneBank (NCBI) accession number MH059553 [9]. This strain was deposited at the Key Laboratory of Tropical Medicinal Resource Chemistry of Ministry of Education, Hainan Normal University, Haikou, China.

### 3.3. Extraction and Isolation

The fermentation was carried out statically in 45 L of potato glucose liquid medium at 25 °C for one month. The fermented broths were filtered through cheesecloth, and the filtrate was extracted with EtOAc (3 × 45 L, 48 h each). The EtOAc extracts were combined and concentrated under reduced pressure to yield a residue of 64.0 g, which was fractionated by vacuum liquid chromatography (VLC) with a petroleum ether-EtOAc-MeOH gradient, to yield five fractions (Frs. 1−5). Fr. 2 was subjected to a Sephadex LH-20 (300 g) column chromatography (CC) eluting with petroleum ether-CHCl_3_-MeOH (1:1:1, *v*/*v*/*v*), and then further purified by using semi-preparative HPLC (85% MeOH-H_2_O) to give **1** (2.0 mg) and **2** (2.2 mg). Fr. 3 was applied on the VLC to obtain three subfractions: Frs. 3.1−3.3. Compound **3** was obtained from Fr. 3.1 using Sephadex LH-20 CC (petroleum ether/CHCl_3_/MeOH, 2:1:1, *v*/*v*/*v*). Fr. 4 was subjected to a silica gel (200 g) CC and eluted with a step gradient of petroleum ether/EtOAc to obtain four subfractions: Frs. 4.1−4.4. Fr. 4.1 was subjected to a Sephadex LH-20 column (300 g) eluted with CHCl_3_: MeOH (1:1) and then semi-preparative HPLC (65% MeOH/H_2_O) to yield **5** (3.0 mg) and **6** (5.5 mg). Fr. 4.2.4 was isolated from Fr. 4.2 by reversed-phase silica gel CC, and then purified by HPLC (75% MeOH/H_2_O) to give **4** (4.6 mg) and **7** (3.7 mg). Fr. 4.3 was further separated by reversed-phase silica gel (50 g) CC and eluted with MeOH/H_2_O gradients from 50:50 to 100:0 (*v*/*v*) into three subfractions Frs. 4.3.1−4.3.3. Fr. 4.3.2 was purified by HPLC (55% MeOH/H_2_O) to yeild **8** (3.5 mg), **9** (4.3 mg), and **10** (4.5 mg).

*8-O-methylnodulisporin F* (**1**): white amorphous powder; [*α*]^25^_D_ = −17.8° (*c* 0.18, MeOH); UV (MeOH) *λ*_max_ (log *ε*) 210 (2.31), 228 (2.42); IR (KBr) *ν*_max_ 3261, 1636, 1270 cm^−1^; CD (*c* 0.06, MeOH) *λ*_max_ (Δ*ε*) 212 (+0.6), 239 (−1.2) nm. ^1^H and ^13^C NMR data see Table 1; [+]-HR-ESI-MS *m*/*z*: 357.1693 [M + H]^+^ (calcd. for C_21_H_25_O_5_ 357.1697).

*Nodulisporin H* (**2**): white amorphous powder; [*α*]^25^_D_ = +10.6° (*c* 0.26, MeOH); UV (MeOH) *λ*_max_ (log *ε*) 217 (3.21), 260 (2.12); IR (KBr) *ν*_max_ 3259, 1620, 1256 cm^−1^; CD (*c* 0.1, MeOH) *λ*_max_ (Δ*ε*) 216 (−3.0), 256 (−0.7) nm. ^1^H and ^13^C NMR data see Table 1; [+]-HR-ESI-MS *m*/*z*: 357.1693 [M + H]^+^ (calcd. for C_21_H_25_O_5_ 357.1697).

*5-Hydroxy-2-methoxy-6,7-dimethyl-1,4-naphthoquinone* (**3**): yellow powder; UV (MeOH) *λ*_max_ 282 (4.20), 249 (3.40), 217 (3.26) nm; IR (KBr) *ν*_max_ 3240, 1865, 1680 cm^−1^; ^1^H and ^13^C NMR data see Table 2; [+]-HR-ESI-MS *m*/*z*: 233.0809 [M + H]^+^ (C_13_H_13_O_4_, calcd. for 233.0808).

*5-Hydroxy-2-methoxynaphtho [9–c] furan-1,4-dione* (**4**): brown powder; UV (MeOH) *λ*_max_ 301 (3.80), 250 (3.12), 217 (2.90) nm; IR (KBr) *ν*_max_ 3246, 1864, 1680 cm^−1^; ^1^H and ^13^C NMR data see Table 2; [+]-HR-ESI-MS *m*/*z*: 247.0605 [M + H]^+^ (C_13_H_11_O_5_, calcd. for 247.0601).

*1*,*3*,*8-Trimethoxynaphtho [9–c] furan* (**5**): yellow powder; UV (MeOH) *λ*_max_ 278 (3.60) nm; IR (KBr) *ν*_max_ 3500, 1632 cm^−1^; ^1^H and ^13^C NMR data see Table 2; [+]-HR-ESI-MS *m*/*z*: 261.1118 [M + H]^+^ (C_15_H_17_O_4_, calcd. for 261.1121).

### 3.4. Biological Assay

The *α*-glucosidase inhibitory activity of **1**–**10** was determined using the procedure reported by Sawada et al. [22], with modifications for carrying out in 96-well plates, and acarbose was used as a positive control.

Compounds **1**–**10** were assayed against four terrestrial pathogenic bacteria *Staphylococcus aureus* (ATCC 25923), methicillin-resistant *S. aureus* MRSA (ATCC 33591), *Escherichia coli* (ATCC 25922), and *Bacillus cereus* (ATCC 11778), and two marine pathogenic bacteria *Vibrio parahaemolyticus* (ATCC 17802) and *V. alginolyticus* (ATCC 17749), and the tests were performed as previously described [23]. Ciprofloxacin was used as the positive control.

## 4. Conclusions

From the mangrove-derived fungus *Daldinia eschscholtzii* HJ004, two new polyketides 8-*O*-methylnodulisporin F (**1**) and nodulisporin H (**2**), two new naphthoquinones 5-hydroxy-2-methoxy-6,7-dimethyl-1,4-naphthoquinone (**3**), 5-hydroxy-2-methoxy-naphtho [9–*c*] furan-1,4-dione (**4**), and a new naphthofuran 1,3,8-trimethoxynaphtho [9–*c*] furan (**5**), and five known compounds (**6**–**10**) were obtained. Compounds **4** and **7** displayed potent inhibitory activity against *α*-glucosidase with the IC_50_ values of 5.7 and 1.1 μg/mL, respectively. Compounds **1** and **2** showed moderate antibacterial activity against *S. aureus*, Methicillin-resistant *S. aureus* MRSA and *B. cereus* with MIC values ranging from 6.25 to 12.5 μg/mL. Compound **3** exhibited antibacterial activity against *B. cereus* with the MIC value of 12.5 μg/mL.

## Figures and Tables

**Table 1:** ^1^H and ^13^C NMR spectroscopic data (400 and 100 MHz) for **1** and **2** in CDCl_3_.

Position	1		2	
	*δ*_C_, Type	*δ*_H_ (*J* in Hz)	*δ*_C_, Type	*δ*_H_ (*J* in Hz)
1	126.0, C		125.7, C	
2	135.3, CH	6.64, d (8.0)	134.0, CH	6.90, d (8.4)
3	105.4, CH	6.07, d (8.0)	107.6, CH	6.22, d (8.4)
4	156.2, C		158.8, C	
5	208.1, C		208.1, C	
6	46.8, CH_2_	3.12, t (7.2)	46.9, CH_2_	3.10, dd (7.8, 6.6)
7	17.9, CH_2_	1.75, m	18.0, CH_2_	1.73, m
8	14.1, CH_3_	1.01, t (7.4)	14.0, CH_3_	0.99, t (7.4)
9	161.0, C		158.3, C	
10	111.7, C		110.2, C	
1′	29.5, CH	4.54, dd (5.2, 0.8)	32.0, CH_2_	4.44, dd (10.4, 8.0)
2′	34.5, CH_2_	1.89, ddd (14.0, 11.6, 5.2)2.07, dt (14.0, 2.0)	39.1, CH_2_	1.77, ddd (14.0, 11.2, 10.4)2.39, ddd (14.0, 8.0, 1.6)
3′	67.9, CH	3.98, ddq (11.6, 6.4, 2.0)	72.6, CH	4.09, ddq (11.2, 6.4, 1.6)
5′	109.7, CH	6.55, brd (8.0)	110.5, CH	6.58, br d (8.0)
6′	128.1, CH	7.14, dd (8.0, 7.8)	127.8, CH	7.11, dd (8.0, 7.8)
7′	102.2, CH	6.42, d (7.8)	103.8, CH	6.42, br d (7.8)
8′	158.1, C		158.6, C	
9′	156.8, C		158.0, C	
10′	109.5, C		114.0, C	
3′-Me	21.5, CH_3_	1.30, d (6.4)	21.3, CH_3_	1.38, d (6.4)
8′-OMe	55.8, CH_3_	3.63, s	55.7, CH_3_	3.48, s
9-OH		13.1, s		

**Table 2:** ^1^H and ^13^C NMR spectroscopic data (400 and 100 MHz) for **3**–**5** in CDCl_3_.

Position	3		4		5	
	*δ*_C_, Type	*δ*_H_ (*J* in Hz)	*δ*_C_, Type	*δ*_H_ (*J* in Hz)	*δ*_C_, Type	*δ*_H_ (*J* in Hz)
1	179.8, C		179.4, C		157.5, C	
2	161.2, C		161.3, C		99.0, CH	6.71, d (2.2)
3	109.5, CH	6.05, s	109.5, CH	6.08, s	158.1, C	
4	190.9, C		191.0, C		98.8, CH	6.51, d (2.2)
4a	111.7, C		113.9, C		139.0, C	
5	159.7, C		156.0, C		114.5, CH	7.30, s
6	134.2, C		134.7, C		127.7, C	
7	145.1, C		147.9, C		140.5, C	
8	121.4, CH	7.50, s	112.6, CH	7.56, s	150.6, C	
8a	159.7, C		131.8, C		115.4, C	
9	11.9, CH_3_	2.25, s	72.1, CH_2_	5.20, s	73.3, CH_2_	5.16, s
10	20.8, CH_3_	2.36, s	74.3, CH_2_	5.16, s	71.7, CH_2_	5.26, s
1-OMe					56.2, CH_3_	3.97, s
2-OMe	56.7, CH_3_	3.90, s	56.9, CH_3_	3.93, s		
3-OMe					55.4, CH_3_	3.89, s
8-OMe					61.6, CH_3_	3.84, s
5-OH		12.61, s		12.34, s		

**Table 3:** Antibacterial activity for **1**–**3**.

Compound	MIC (μg/mL)		
	*S. aureus*	MRSA	*B. cereus*
**1**	6.25	12.5	6.25
**2**	12.5	12.5	6.25
**3**	>25	>25	12.5
Ciprofloxacin *^a^*	0.31	1.25	1.25

Ciprofloxacin *^a^* was used as a positive control.

## Supplementary Materials

The following are available online at https://www.mdpi.com/1660-3397/17/12/710/s1, NMR and MS data of new compounds **1**–**5**.
