# Supplementary material for: Bioactive Secondary Metabolites from the Culture of the Mangrove-Derived Fungus Daldinia eschscholtzii HJ004
Source: Mar Drugs. 2019 Dec 17;17(12):710. doi: 10.3390/md17120710 (PMC6950716; doi:10.3390/md17120710)
Supplement: Supplementary file 1 [file marinedrugs-17-00710-s001.pdf]

Supporting Information

Bioactive Secondary Metabolites from the Culture of the Mangrove-Derived Fungus *Daldinia eschscholtzii* HJ004

Hai-Xia Liao [1,2,3,4], Tai-Ming Shao [4], Rong-Qing Mei [1,2], Guo-Lei Huang [1,2], Xue-Ming Zhou [1,2], Cai-Juan Zheng [1,2,*] and Chang-Yun Wang [3,*]

1 Key Laboratory of Tropical Medicinal Resource Chemistry of Ministry of Education, Hainan Normal University, Haikou 571158, China; m17864275062@163.com (H.-X.L.); 15707973425@163.com (R.-Q.M.); tianleilei526@163.com (G.-L.H.); xueming2009211@126.com (X.-M.Z.)
2 Key Laboratory of Tropical Medicinal Plant Chemistry of Hainan Province, College of Chemistry and Chemical Engineering, Hainan Normal University, Haikou 571158, China
3 Laboratory for Marine Drugs and Bioproducts, Qingdao National Laboratory for Marine Science and Technology, Qingdao 266071, China
4 Guangxi Key Laboratory of Agricultural Resources Chemistry and Biotechnology, College of Chemistry and Food Science, Yulin Normal University, Yulin 537000, China; shaotm1689@163.com
* Correspondence: caijuan2002@163.com (C.-J.Z.); changyun@ouc.edu.cn (C.-Y.W.)

Content

Figure S1. ^{1}H NMR spectrum of **1** in $CDCl_3$
Figure S2. ^{13}C NMR spectrum of **1** in $CDCl_3$
Figure S3. 135-DEPT spectrum of **1** in $CDCl_3$
Figure S4. HMQC spectrum of **1** in $CDCl_3$
Figure S5. ^{1}H-^{1}H COSY spectrum of **1** in $CDCl_3$
Figure S6. HMBC spectrum of **1** in $CDCl_3$
Figure S7. NOESY spectrum of **1** in $CDCl_3$
Figure S8. HR-ESI-MS spectrum of **1**
Figure S9. ^{1}H NMR spectrum of **2** in $CDCl_3$
Figure S10. ^{13}C NMR spectrum of **2** in $CDCl_3$
Figure S11. 135-DEPT spectrum of **2** in $CDCl_3$
Figure S12. HMQC spectrum of **2** in $CDCl_3$
Figure S13. ^{1}H-^{1}H COSY spectrum of **2** in $CDCl_3$
Figure S14. HMBC spectrum of **2** in $CDCl_3$
Figure S15. NOESY spectrum of **2** in $CDCl_3$
Figure S16. HR-ESI-MS spectrum of **2**
Figure S17. ^{1}H NMR spectrum of **3** in $CDCl_3$
Figure S18. ^{13}C NMR spectrum of **3** in $CDCl_3$
Figure S19. HMQC spectrum of **3** in $CDCl_3$
Figure S20. HMBC spectrum of **3** in $CDCl_3$
Figure S21. HR-ESI-MS spectrum of **3**
Figure S22. ^{1}H NMR spectrum of **4** in $CDCl_3$
Figure S23. ^{13}C NMR spectrum of **4** in $CDCl_3$
Figure S24. HMQC spectrum of **4** in $CDCl_3$
Figure S25. HMBC spectrum of **4** in $CDCl_3$
Figure S26. HR-ESI-MS spectrum of **4**
Figure S27. ^{1}H NMR spectrum of **5** in $CDCl_3$
Figure S28. ^{13}C NMR spectrum of **5** in $CDCl_3$
Figure S29. HMQC spectrum of **5** in $CDCl_3$
Figure S30. HMBC spectrum of **5** in $CDCl_3$
Figure S31. HR-ESI-MS spectrum of **5**

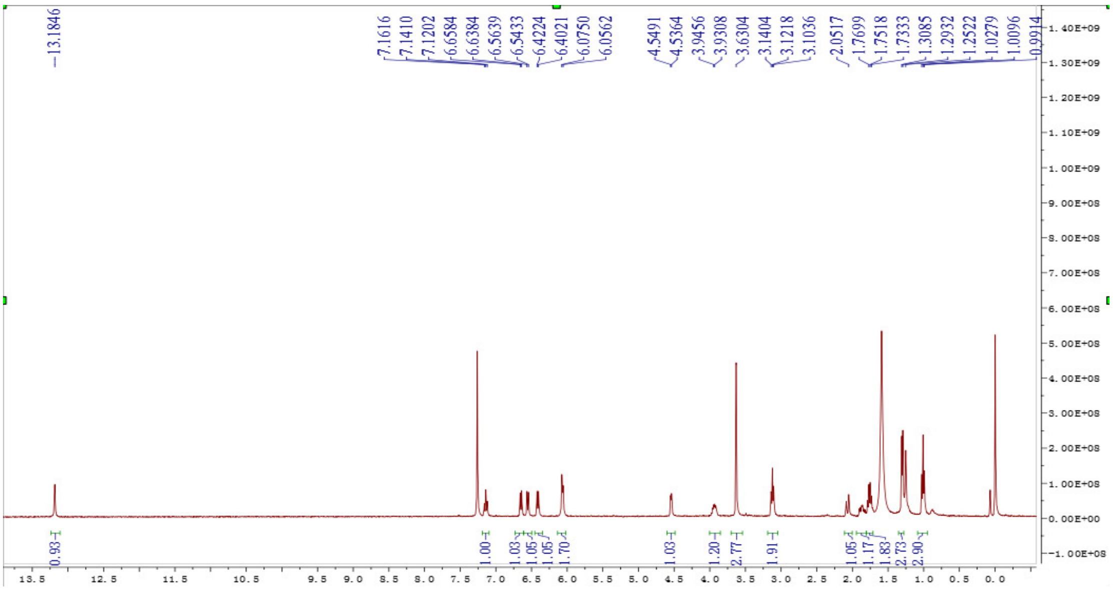

Figure S1. ^{1}H NMR spectrum of **1** in $CDCl_3$

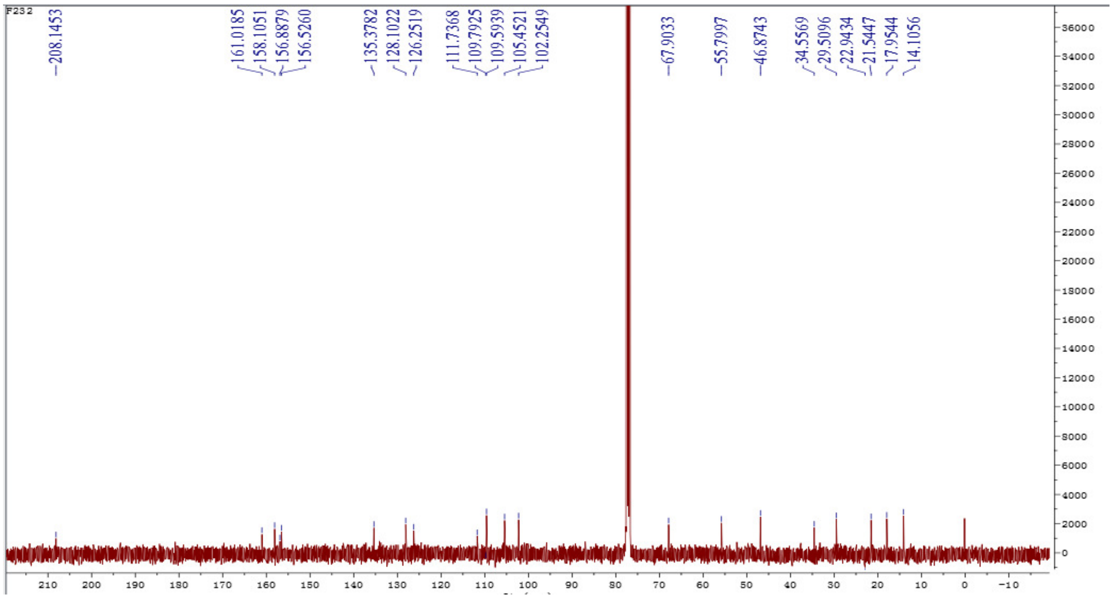

Figure S2. ^{13}C NMR spectrum of **1** in $CDCl_3$

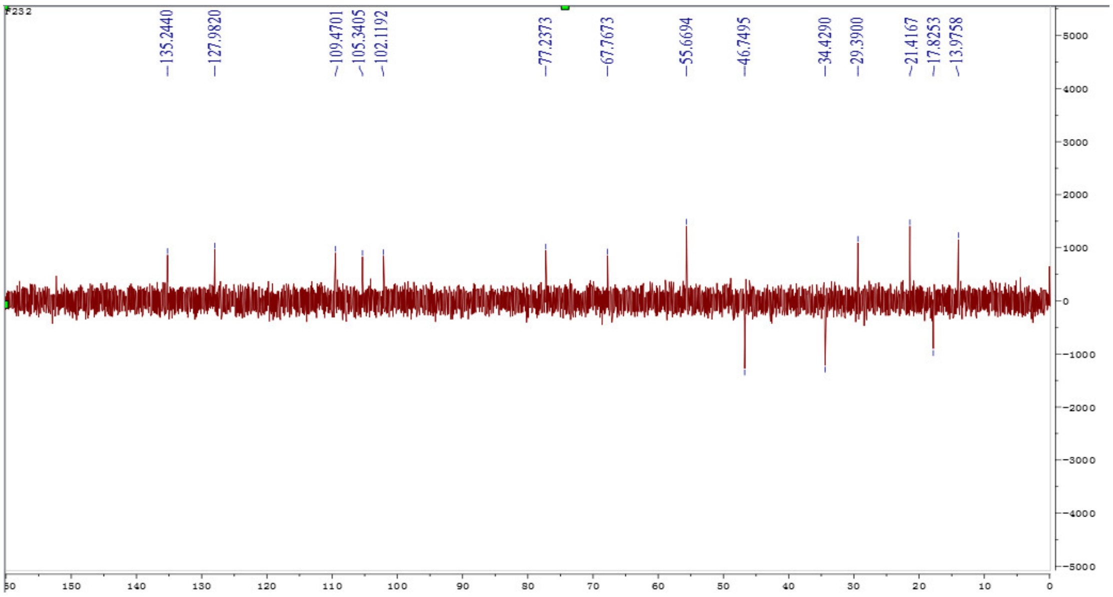

Figure S3. 135-DEPT spectrum of **1** in $CDCl_3$

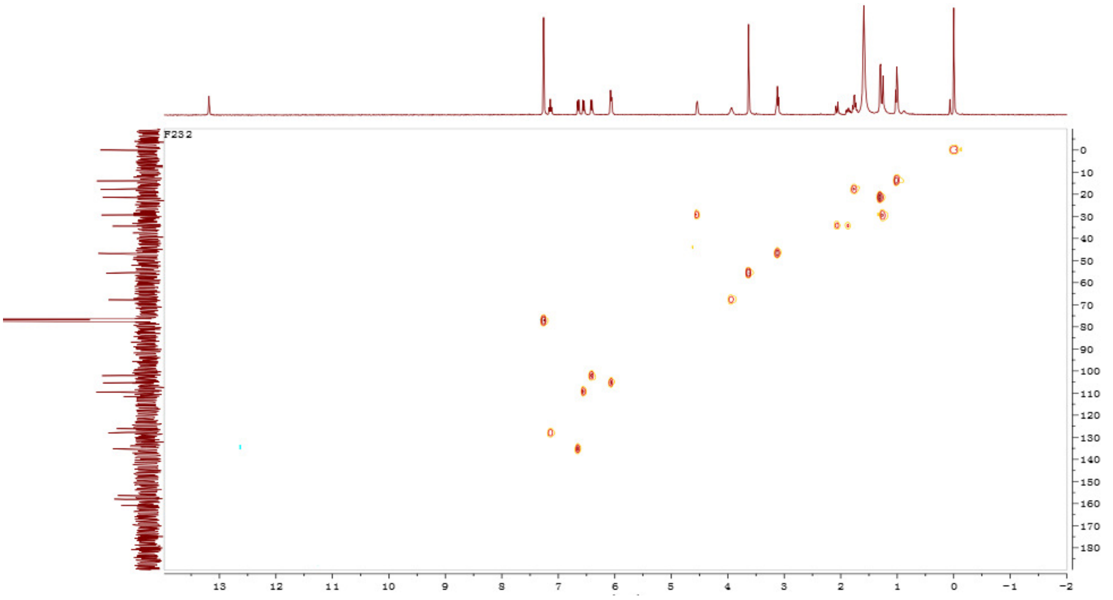

Figure S4. HMQC spectrum of **1** in $CDCl_3$

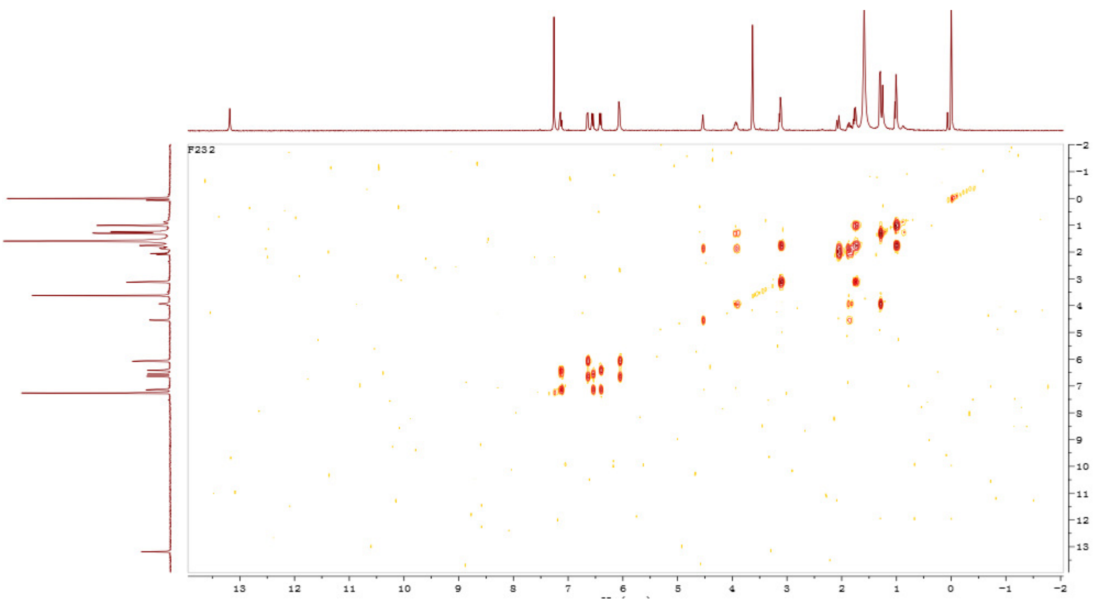

Figure S5. ^{1}H-^{1}H COSY spectrum of **1** in $CDCl_3$

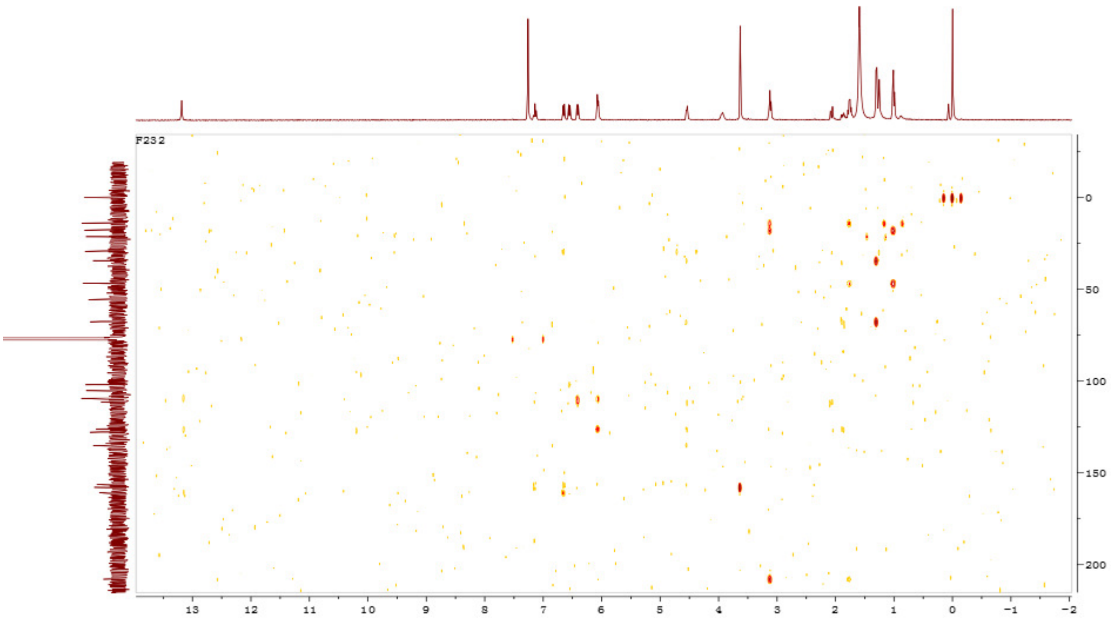

Figure S6. HMBC spectrum of **1** in $CDCl_3$

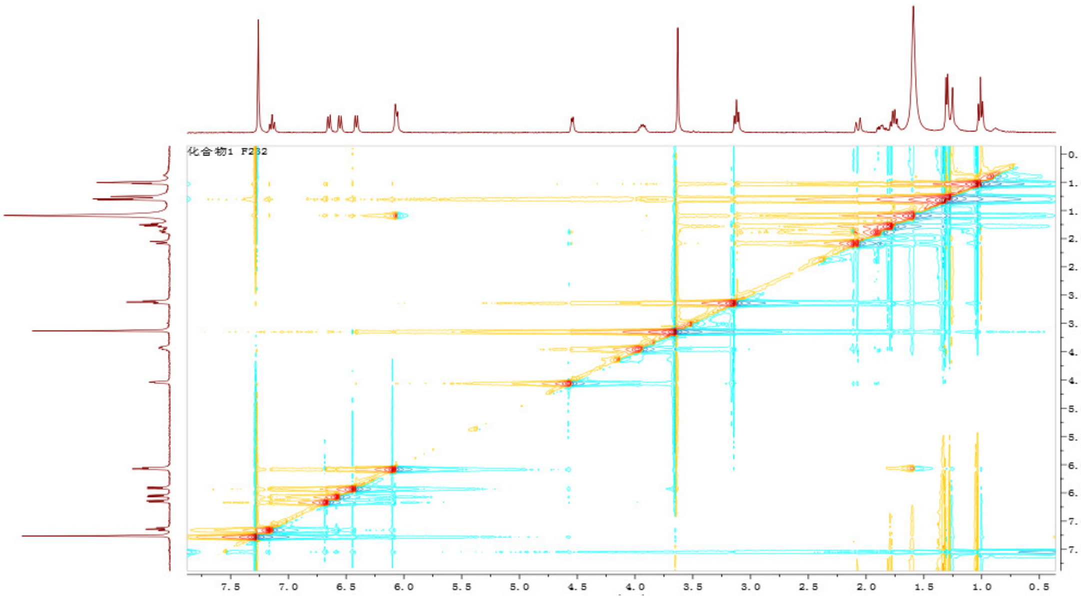

Figure S7. NOESY spectrum of **1** in $CDCl_3$

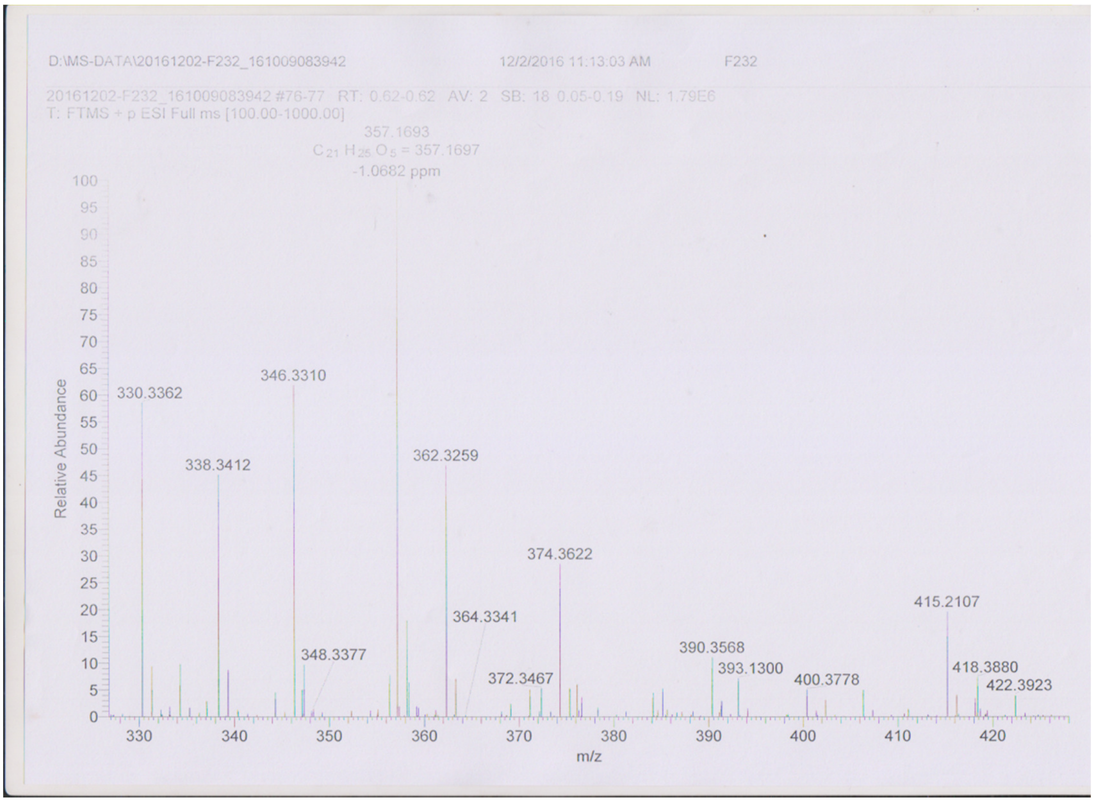

Figure S8. HR-ESI-MS spectrum of **1**

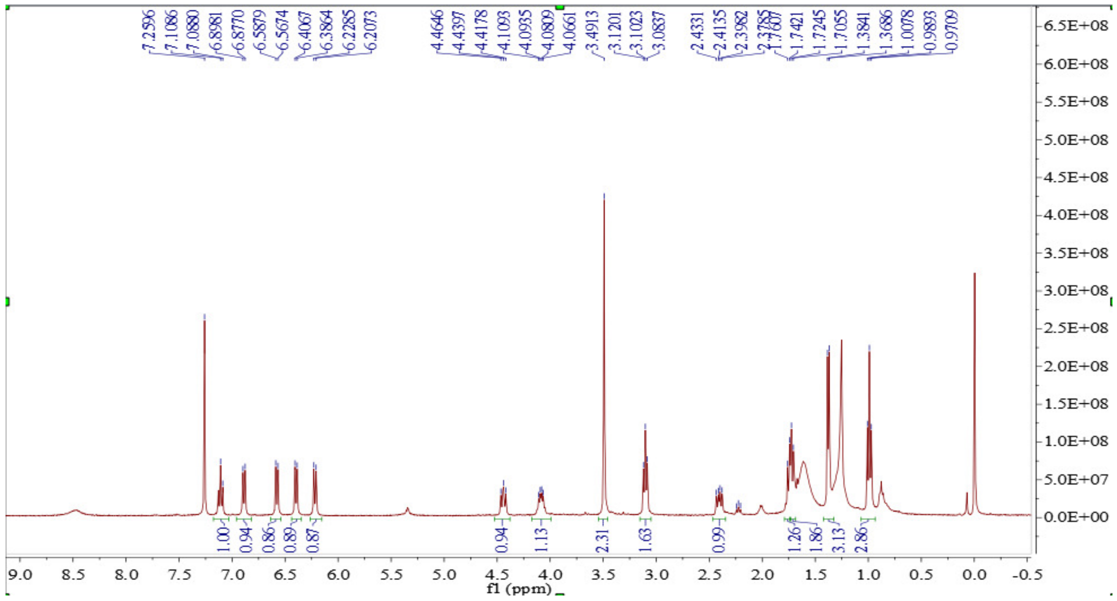

Figure S9. ^{1}H NMR spectrum of **2** in $CDCl_3$

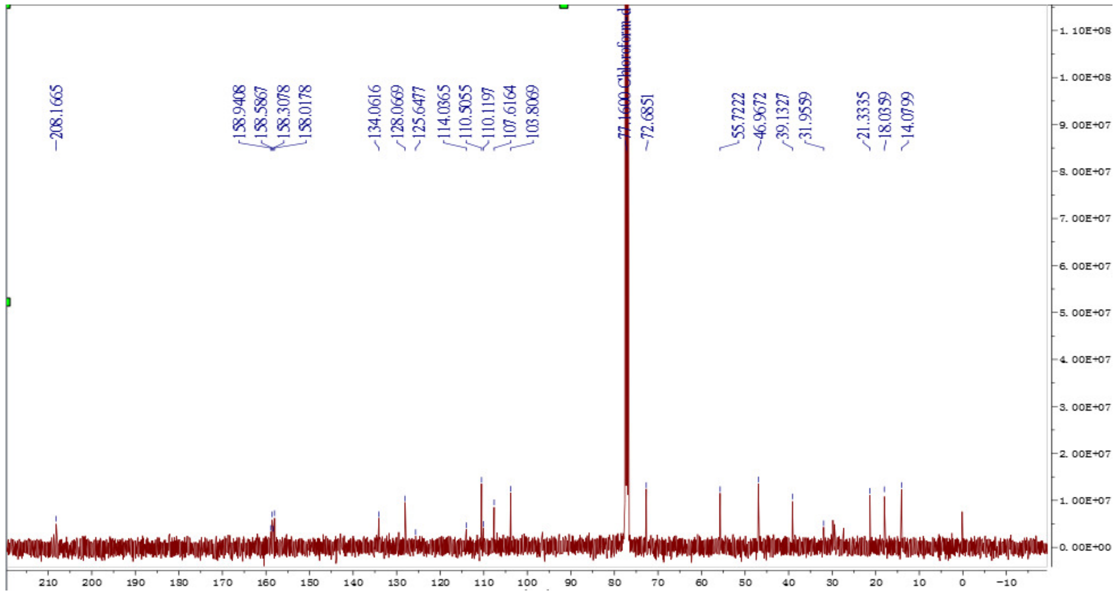

Figure S10. ^{13}C NMR spectrum of **2** in $CDCl_3$

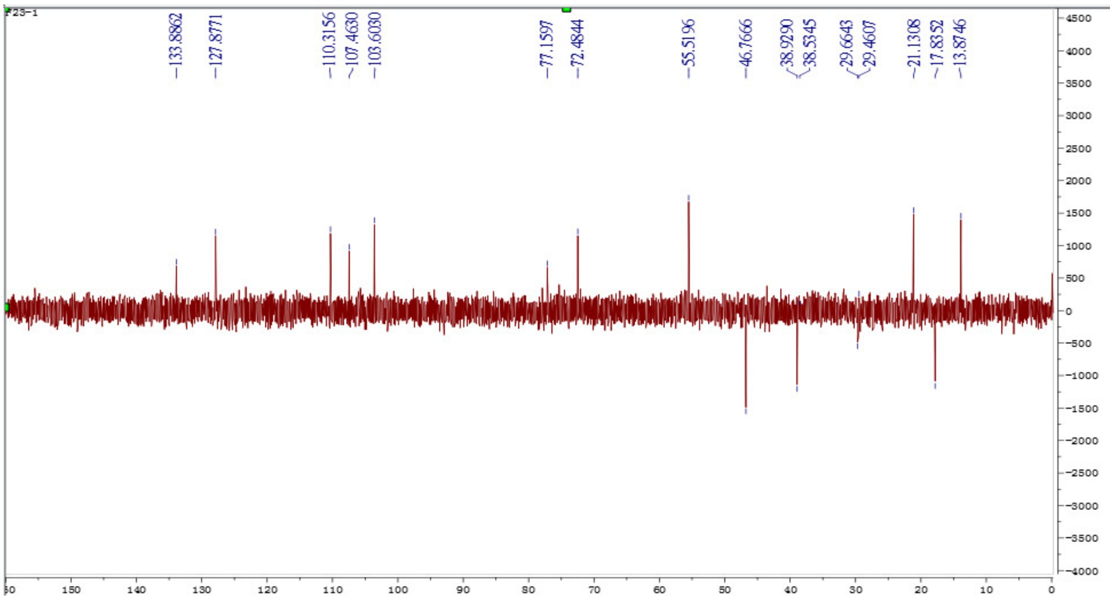

Figure S11. 135-DEPT spectrum of **2** in $CDCl_3$

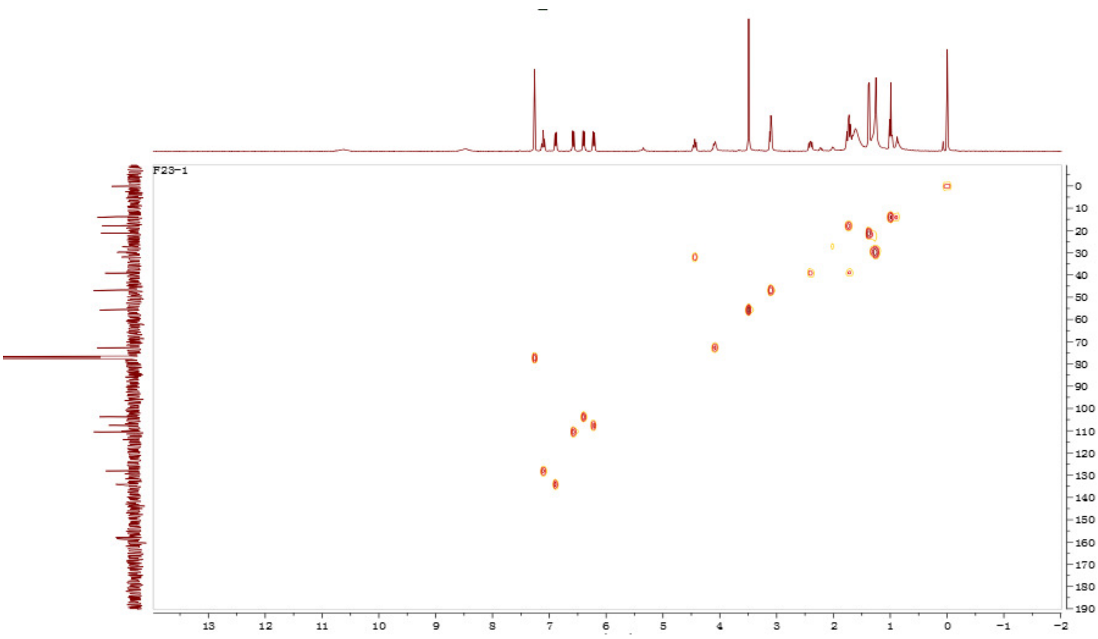

Figure S12. HMQC spectrum of **2** in $CDCl_3$

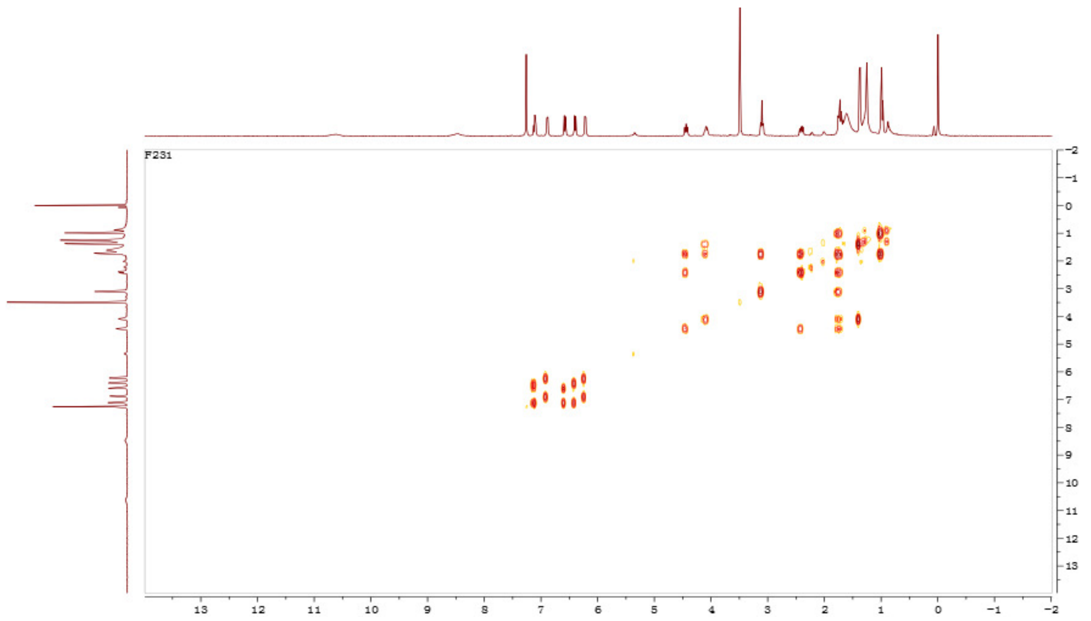

Figure S13. ^{1}H-^{1}H COSY spectrum of **2** in $CDCl_3$

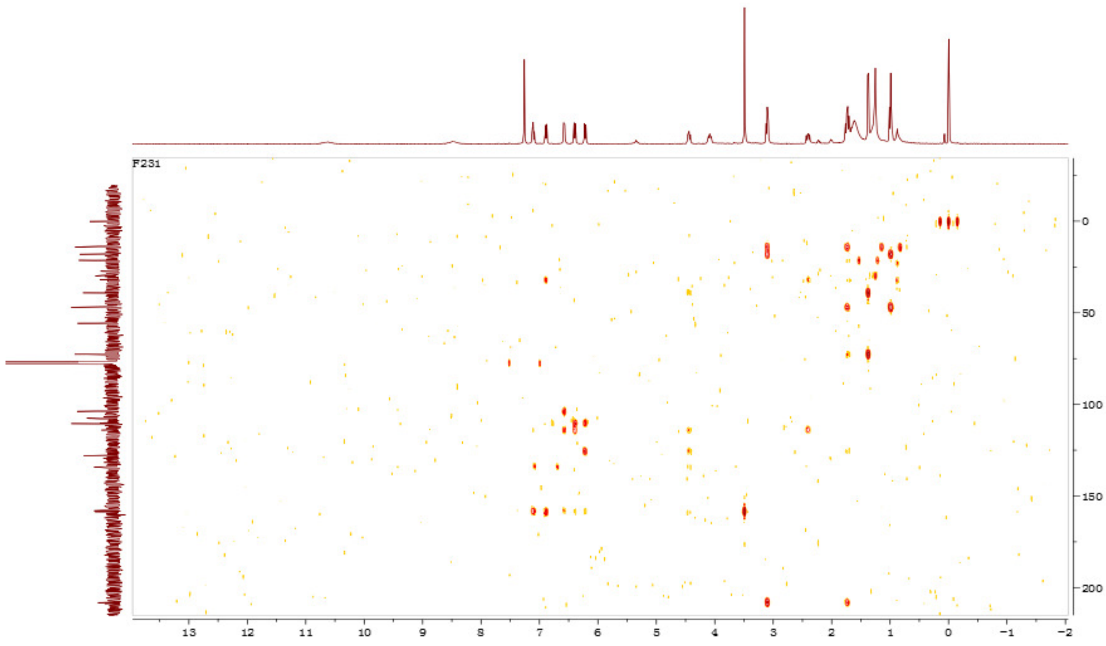

Figure S14. HMBC spectrum of **2** in $CDCl_3$

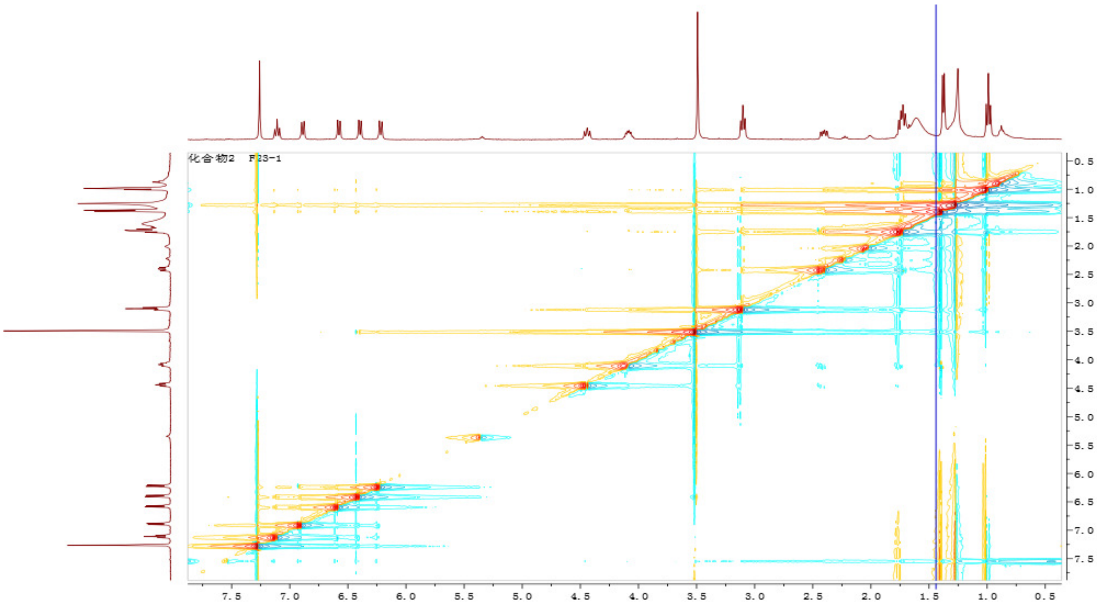

Figure S15. NOESY spectrum of 2 in $CDCl_3$

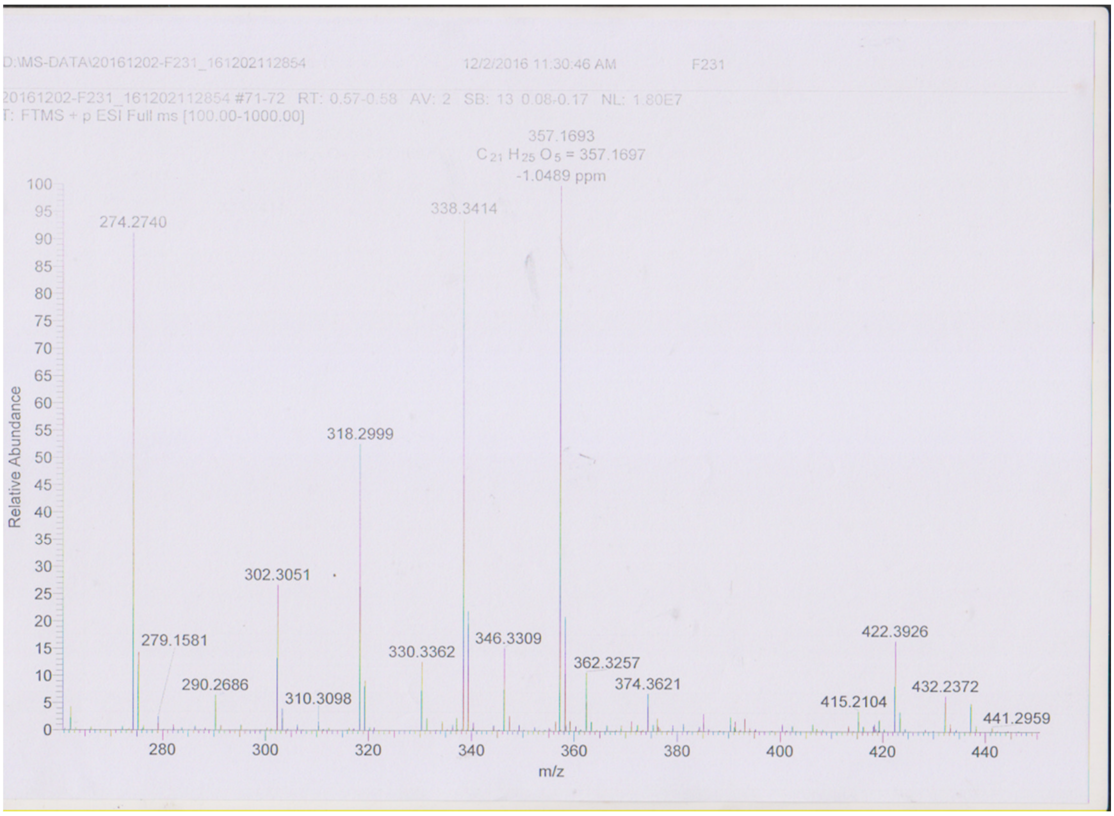

Figure S16. HR-ESI-MS spectrum of **2**

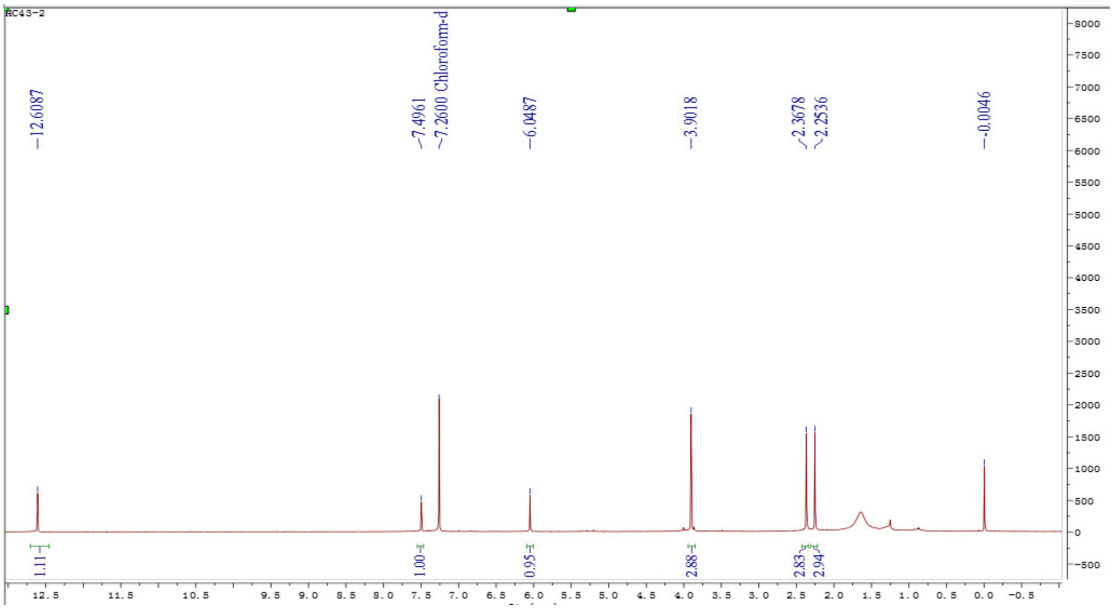

Figure S17. ^{1}H NMR spectrum of **3** in $CDCl_3$

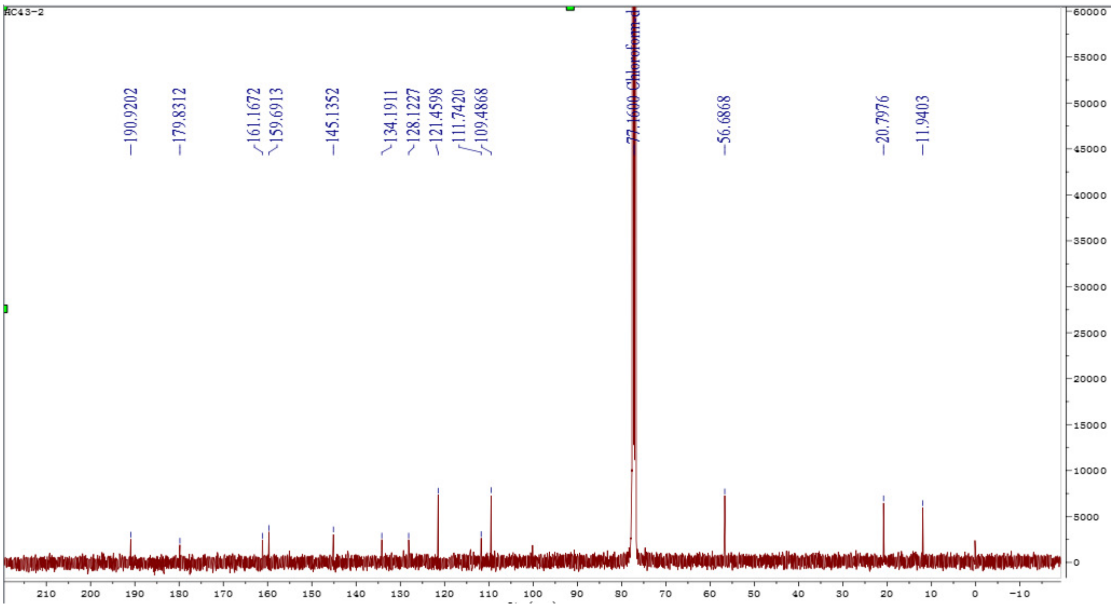

Figure S18. ^{13}C NMR spectrum of **3** in $CDCl_3$

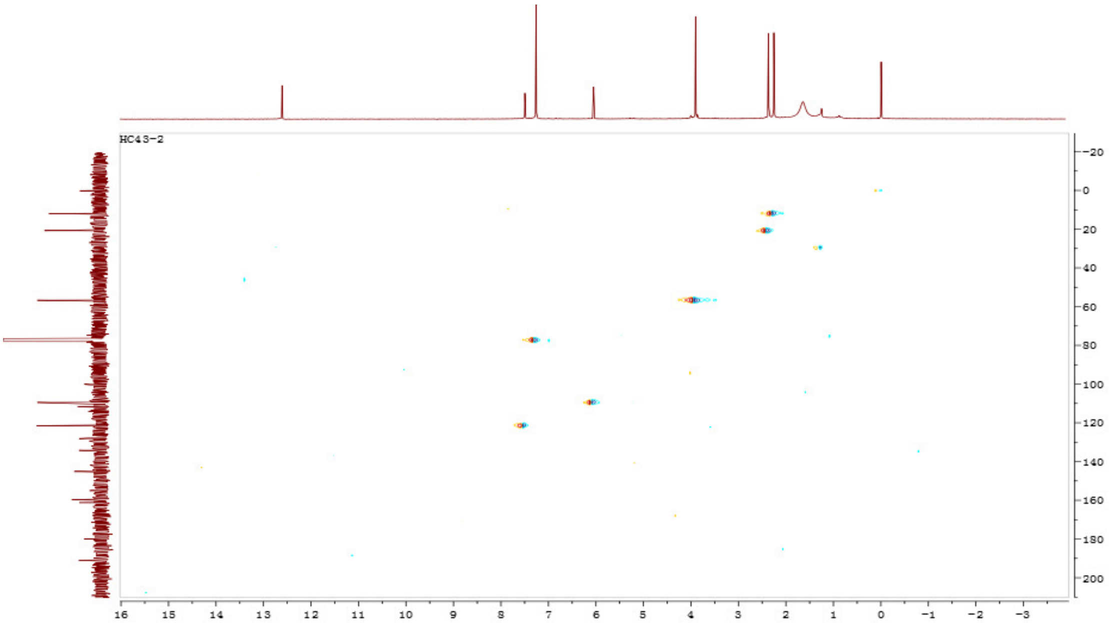

Figure S19. HMQC spectrum of **3** in $CDCl_3$

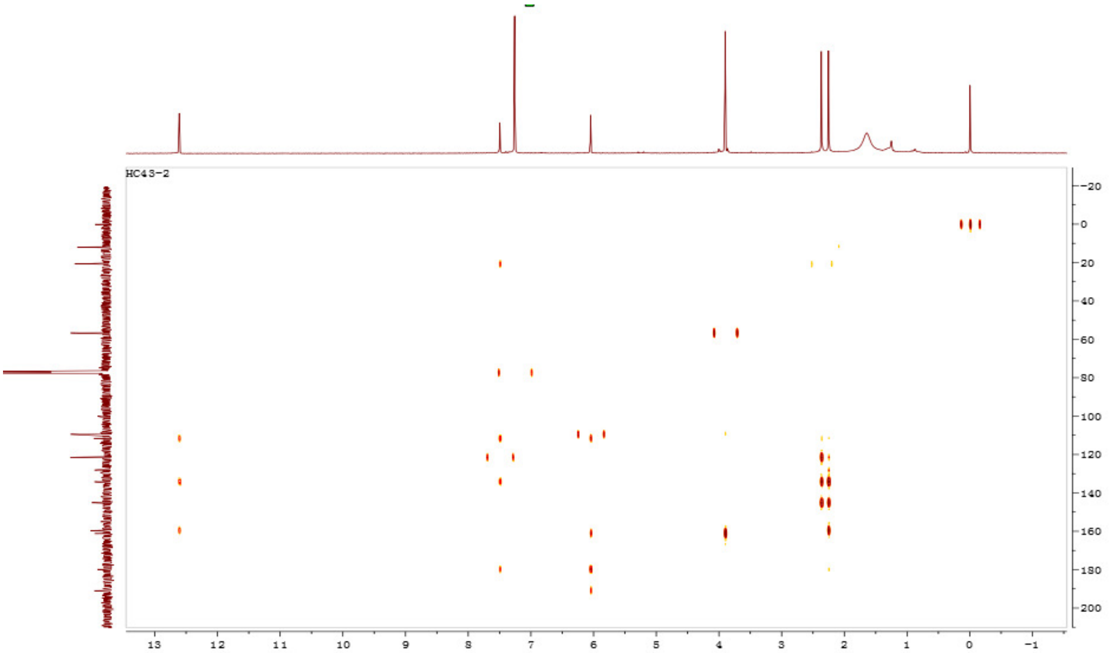

Figure S20. HMBC spectrum of **3** in $CDCl_3$

HC43-2. HRMS (ESI) m/z calcd for $C_{13}H_{13}O_4^+$ $(M+H)^+$ 233.08084, found 233.08087.

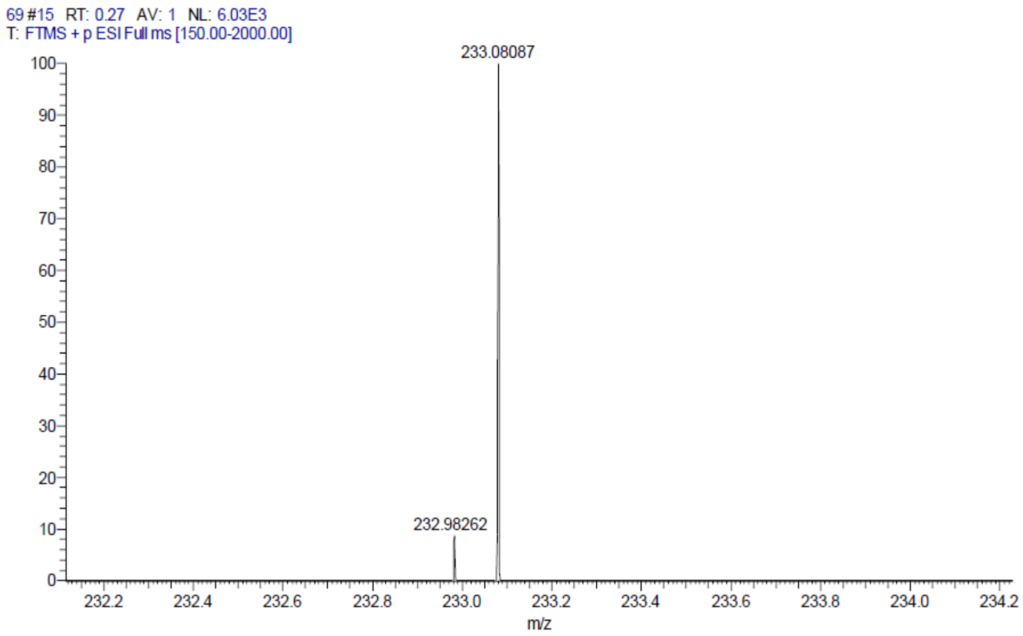

Figure S21. HR-ESI-MS spectrum of **3**

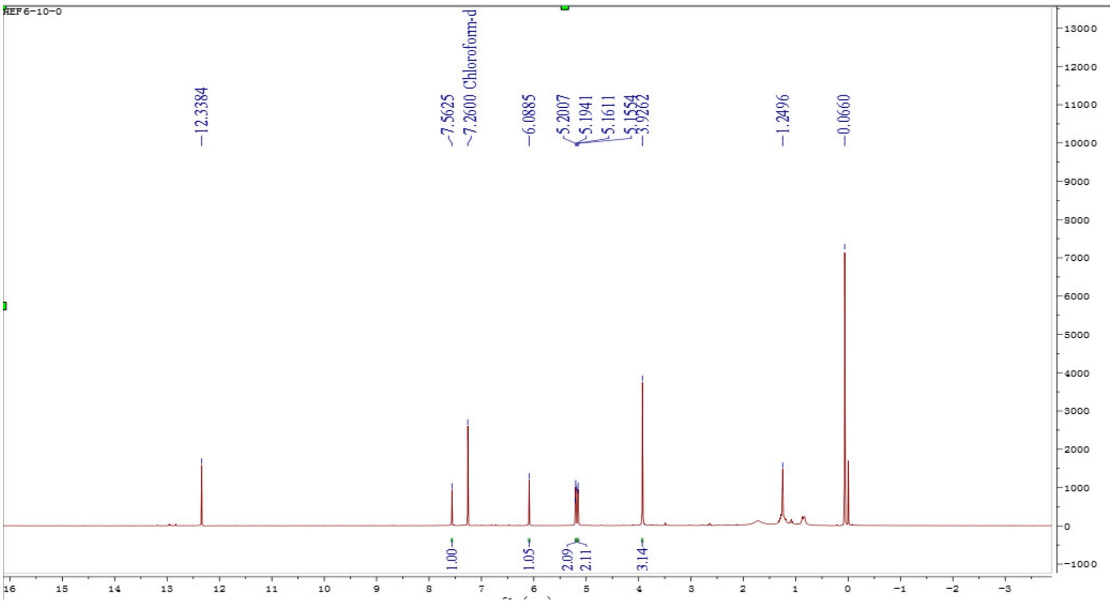

Figure S22. ^{1}H NMR spectrum of **4** in $CDCl_3$

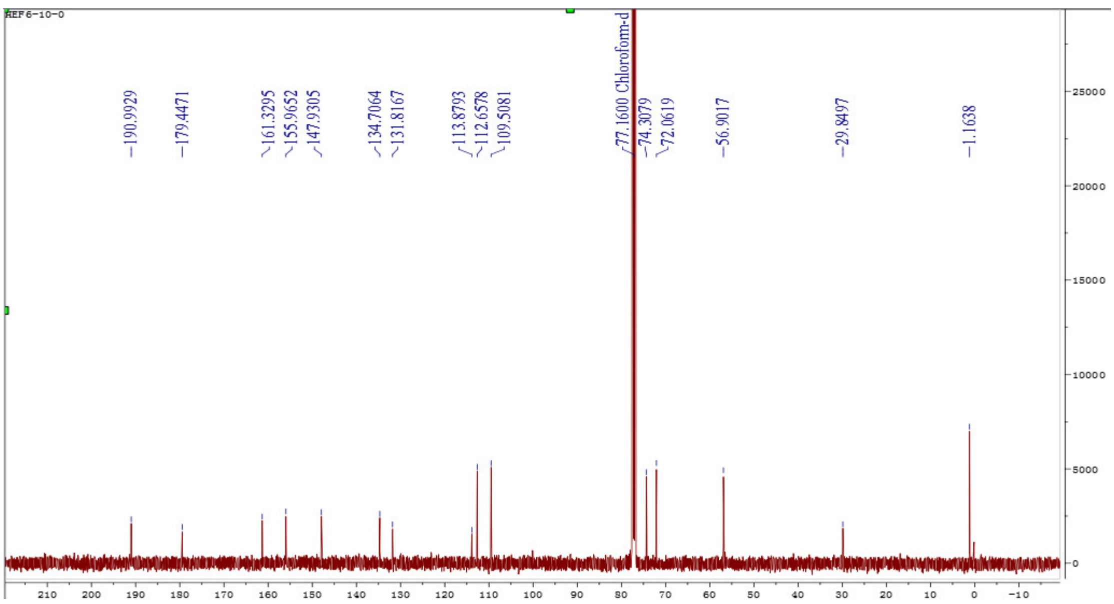

Figure S23. ^{13}C NMR spectrum of **4** in $CDCl_3$

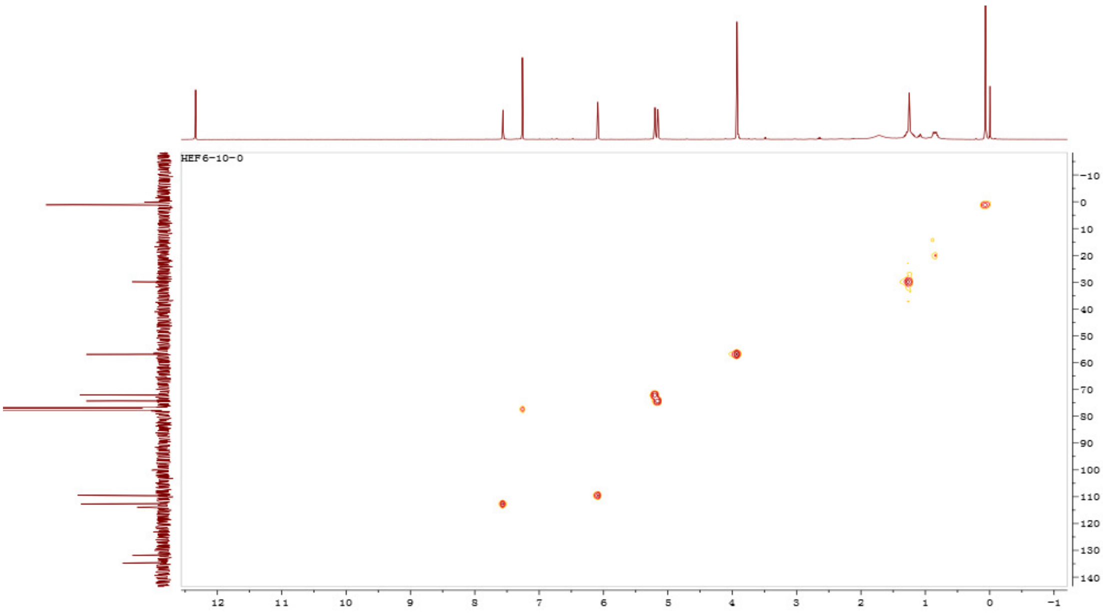

Figure S24. HMQC spectrum of **4** in $CDCl_3$

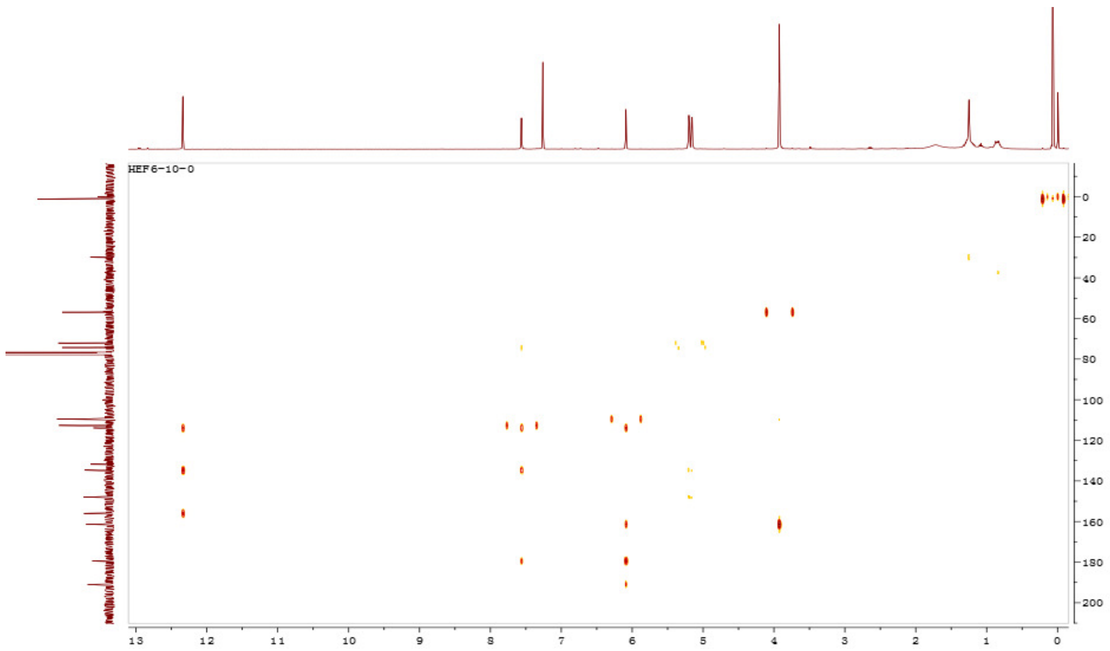

Figure S25. HMBC spectrum of **4** in $CDCl_3$

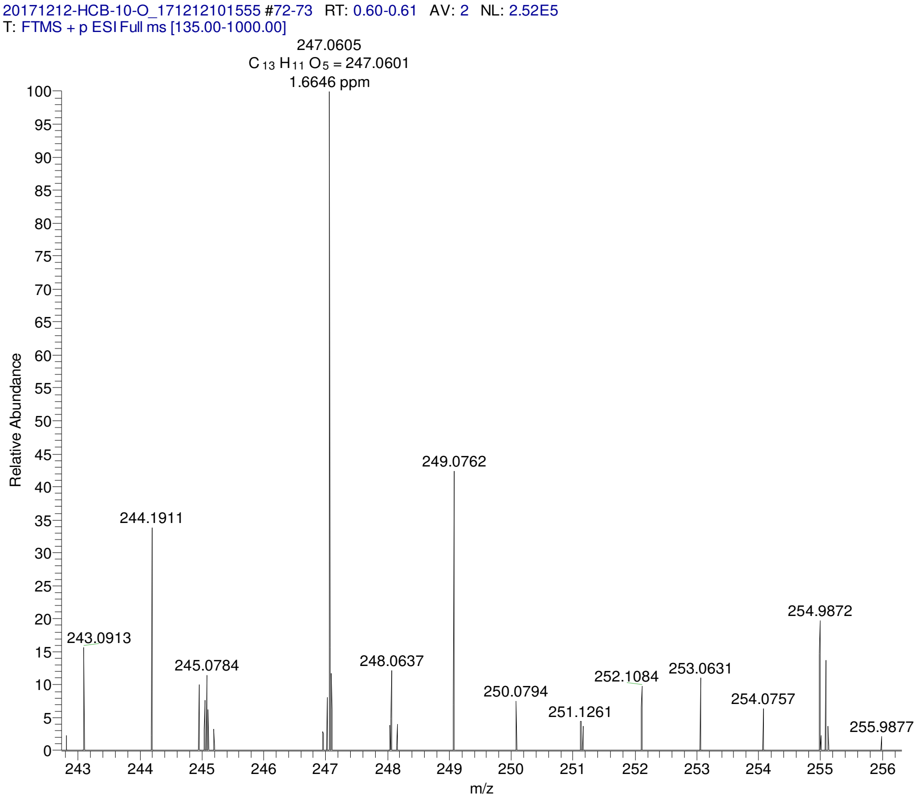

Figure S26. HR-ESI-MS spectrum of **4**

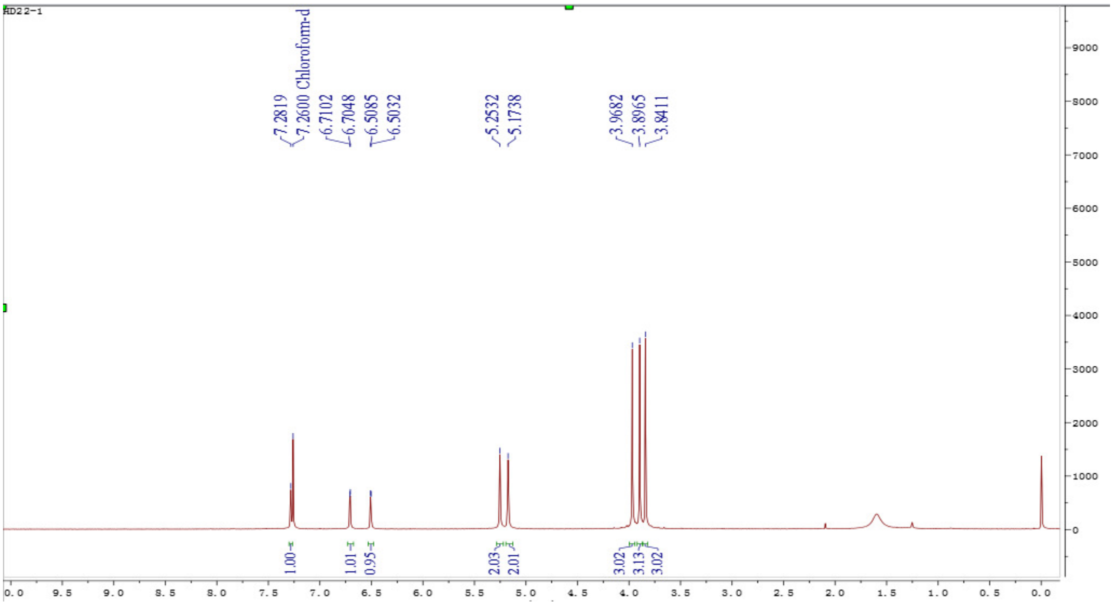

Figure S27. ^{1}H NMR spectrum of **5** in $CDCl_3$

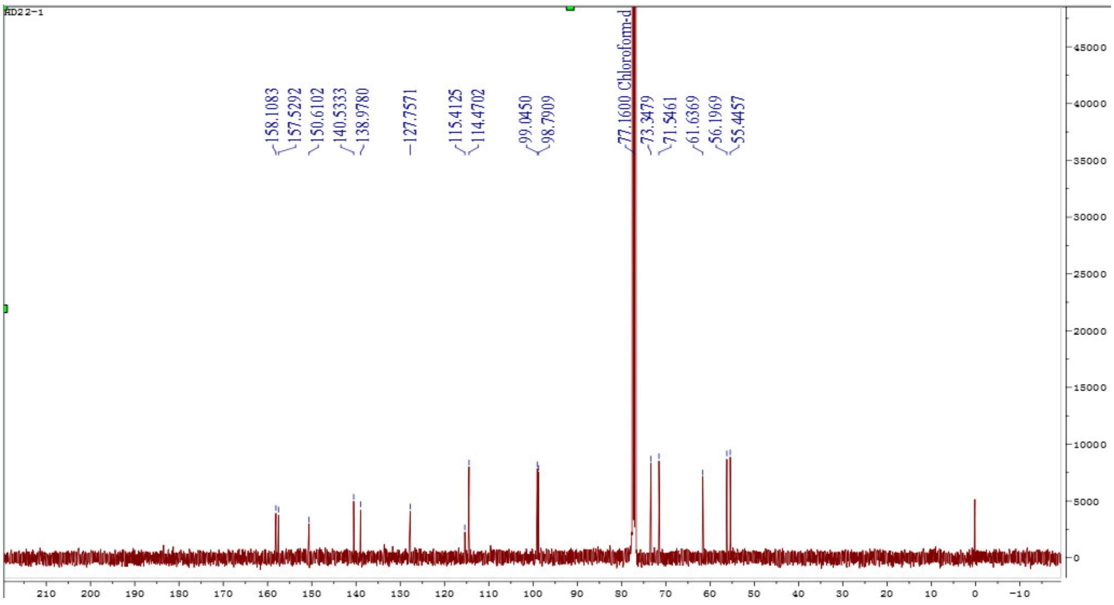

Figure S28. ^{13}C NMR spectrum of **5** in $CDCl_3$

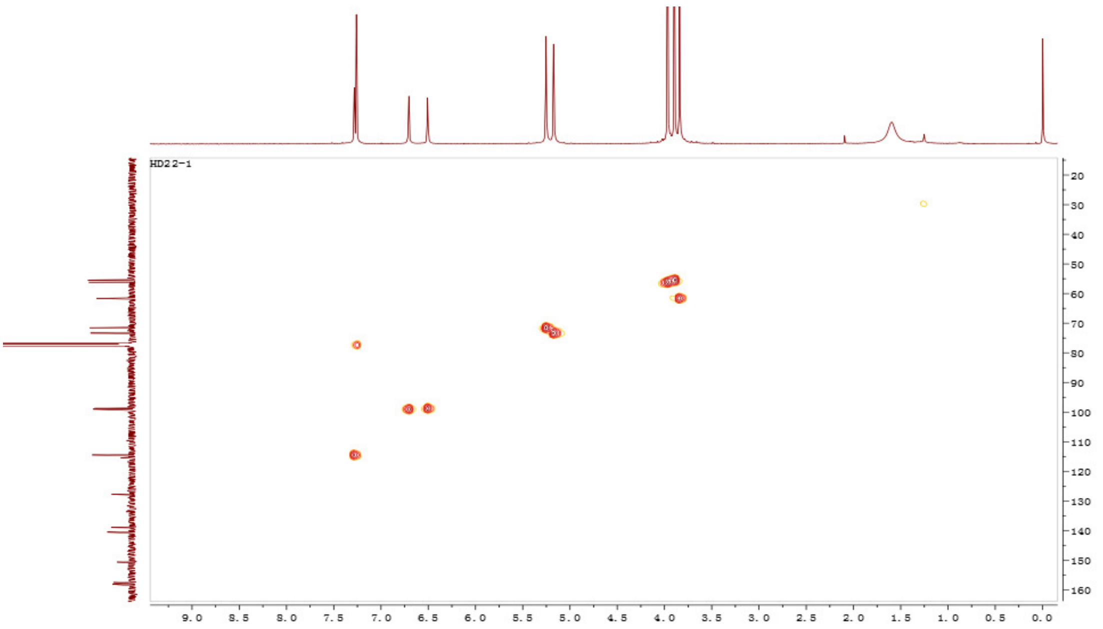

Figure S29. HMQC spectrum of **5** in $CDCl_3$

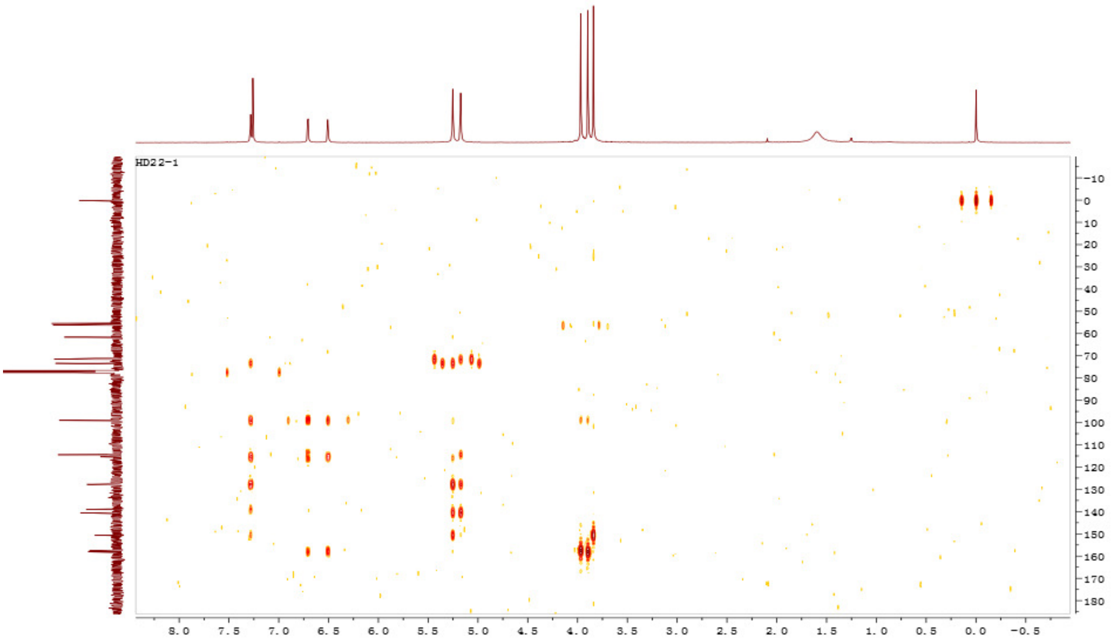

Figure S30. HMBC spectrum of **5** in $CDCl_3$

HD22-1. HRMS (ESI) m/z calcd for $C_{15}H_{17}O_4^+$ $(M+H)^+$ 261.11214, found 261.11185.

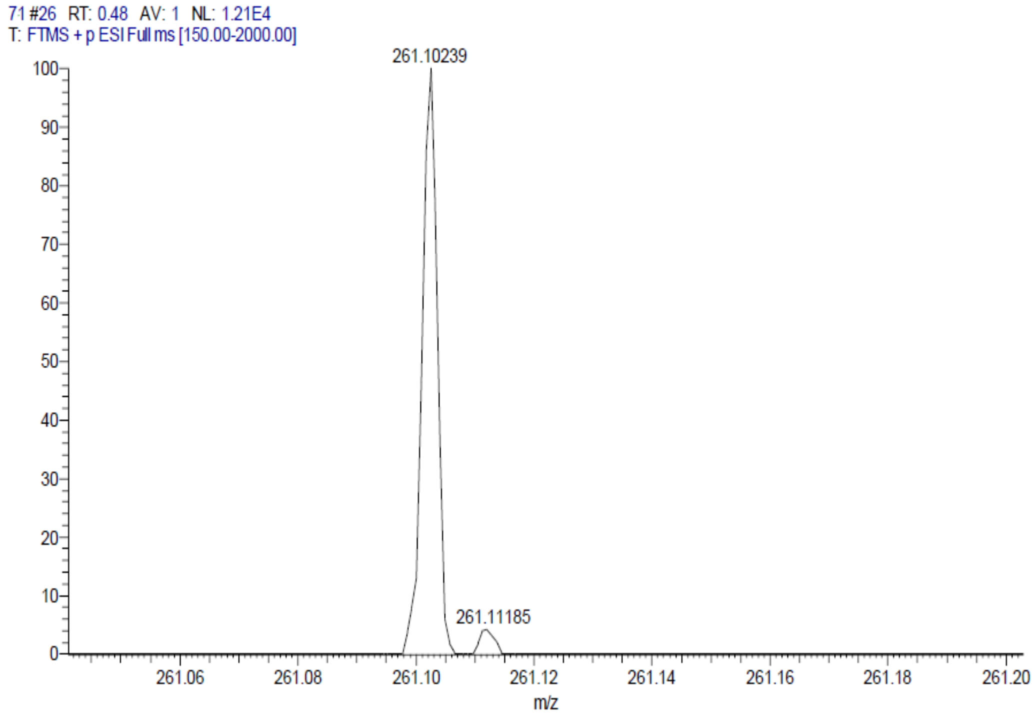

Figure S31. HR-ESI-MS spectrum of **5**
